# The Clinical Impact of Chromosomal Microarray on Paediatric Care in Hong Kong

**DOI:** 10.1371/journal.pone.0109629

**Published:** 2014-10-15

**Authors:** Victoria Q. Tao, Kelvin Y. K. Chan, Yoyo W. Y. Chu, Gary T. K. Mok, Tiong Y. Tan, Wanling Yang, So Lun Lee, Wing Fai Tang, Winnie W. Y. Tso, Elizabeth T. Lau, Anita S. Y. Kan, Mary H. Tang, Yu-lung Lau, Brian H. Y. Chung

**Affiliations:** 1 Department of Paediatrics and Adolescent Medicine, LKS Faculty of Medicine, The University of Hong Kong, Hong Kong Special Administrative Region, China; 2 Department of Obstetrics and Gynecology, Queen Mary Hospital, Hong Kong Special Administrative Region, China; 3 Victorian Clinical Genetics Service, Murdoch Children's Research Institute, Royal Children's Hospital, Department of Paediatrics, University of Melbourne, Melbourne, Australia; 4 Department of Obstetrics and Gynecology, LKS Faculty of Medicine, The University of Hong Kong, Hong Kong Special Administrative Region, China; Emory University School Of Medicine, United States of America

## Abstract

**Objective:**

To evaluate the clinical impact of chromosomal microarray (CMA) on the management of paediatric patients in Hong Kong.

**Methods:**

We performed NimbleGen 135k oligonucleotide array on 327 children with intellectual disability (ID)/developmental delay (DD), autism spectrum disorders (ASD), and/or multiple congenital anomalies (MCAs) in a university-affiliated paediatric unit from January 2011 to May 2013. The medical records of patients were reviewed in September 2013, focusing on the pathogenic/likely pathogenic CMA findings and their “clinical actionability” based on established criteria.

**Results:**

Thirty-seven patients were reported to have pathogenic/likely pathogenic results, while 40 had findings of unknown significance. This gives a detection rate of 11% for clinically significant (pathogenic/likely pathogenic) findings. The significant findings have prompted clinical actions in 28 out of 37 patients (75.7%), while the findings with unknown significance have led to further management recommendation in only 1 patient (p<0.001). Nineteen out of the 28 management recommendations are “evidence-based” on either practice guidelines endorsed by a professional society (n = 9, Level 1) or peer-reviewed publications making medical management recommendation (n = 10, Level 2). CMA results impact medical management by precipitating referral to a specialist (n = 24); diagnostic testing (n = 25), surveillance of complications (n = 19), interventional procedure (n = 7), medication (n = 15) or lifestyle modification (n = 12).

**Conclusion:**

The application of CMA in children with ID/DD, ASD, and/or MCAs in Hong Kong results in a diagnostic yield of ∼11% for pathogenic/likely pathogenic results. Importantly the yield for clinically actionable results is 8.6%. We advocate using diagnostic yield of clinically actionable results to evaluate CMA as it provides information of both clinical validity and clinical utility. Furthermore, it incorporates evidence-based medicine into the practice of genomic medicine. The same framework can be applied to other genomic testing strategies enabled by next-generation sequencing.

## Introduction

Chromosomal microarray (CMA) has emerged as a major tool to identify unbalanced chromosomal aberrations in children for its higher resolution compared to conventional cytogenetics and is recommended as the first-tier investigation for intellectual disability (ID)/developmental delay (DD), autism spectrum disorders (ASD) and multiple congenital anomalies (MCAs). [1]–[9] Balanced rearrangements and low-level mosaicism are generally not detectable; however, these are relatively infrequent causes of abnormal phenotypes in patients (<1%). [2] Large-scale studies in Asian populations have revealed similar detection rates compared to studies conducted in Europe and Northern America. [10]–[13]

While the clinical interpretation of microarray anomalies remains an ongoing challenge, the impact of CMA results on clinical management is not well studied. Surveys of physicians showed changes in management in 70% patients with positive CMA results [14]. Multiple case reports have demonstrated the usefulness of CMA in identifying the genetic causes in patients with unknown diagnoses and in uncovering cancer susceptibility. [15]–[17] In a cohort of 1792 patients with ID, ASD and/or MCAs, management recommendations were made in 54% patients with clinically significant CMA results and 34% with findings of possible significance. [18] Riggs et al. compiled a list of 146 genomic disorders which would be detected by CMA for which there are published evidence supporting management recommendation and identified that 7% of all cases in the ISCA (International Standards for Cytogenomic Arrays) Consortium database are “clinically actionable”. [19] In a review based on 46298 cases in the laboratory database, Ellison et al found that 35% of the cases with positive CMA results were established microdeletion/microduplication syndromes, conditions with increased cancer susceptibility or other actionable conditions associated with dosage-sensitive genes. [20] Henderson et al. found that 55% of the positive CMA results prompted clinical actions in their cohort of 1780 cases. [21]

Despite the growing evidence of its diagnostic yield and cost-effectiveness [22], CMA has not yet been implemented as a first-tier diagnostic test for the above mentioned conditions for children in Hong Kong. The objective of this study is to evaluate the clinical impact of CMA on the medical management of the paediatric patients in whom CMA was applied as first-tier clinical testing in Hong Kong. We study the clinical impact of CMA by evaluating the detection rate of pathogenic/likely pathogenic findings and the proportion of these findings that are clinically actionable, and the level of evidence supporting these recommendations.

## Materials and Methods

### Patients and Samples

From January 2011, we started to offer CMA to paediatric patients in 2 university-affiliated hospitals: Queen Mary Hospital (QMH) and the Duchess of Kent Children's Hospital (DKCH). Indications for CMA included unexplained ID/DD, ASD, or multiple MCAs after review by a clinical geneticist. Clinically recognizable syndromic conditions, e.g. Down syndrome, were confirmed by conventional cytogenetic (e.g. karyotype)/molecular tests (e.g. rapid aneuploidy detection by QF-PCR and fluorescent *in-situ* hybridization, FISH) instead of “first-tiered” CMA, and referred for CMA when conventional investigation showed negative results. Written informed consent for CMA was obtained from all parents/legal guardians. Clinicians or geneticists counseled the parents/guardians about the indication for the CMA, benefits and limitations of the test, methodology, reporting time and possible outcomes upon recruitment. Patients who had received prenatal CMA testing or parents who opted not to receive test result were excluded.

### CMA testing and interpretation

For each patient, 3 ml of peripheral blood in EDTA bottle was sent to Prenatal Diagnostic Laboratory, Tsan Yuk Hospital (TYH). All samples were tested by NimbleGen CGX-135K arrays designed by Signature Genomics (Roche NimbleGen, Inc., Madison, WI, USA) following manufacturer's instructions. The coverage of the array has an average resolution of 140 kb across the genome and 40 kb or less in regions of clinical relevance. It evaluates over 245 known genetic syndromes and over 980 gene regions of functional significance in human development. Data were analyzed by Genoglyphix software (Signature Genomics, Spokane, USA). Genomic coordinates were based on genome build hg18.

Detected copy number variants (CNVs) were systematically evaluated for its clinical significance by comparing the CNVs to information in the Signature Genomics' proprietary Genoglyphix Chromosome Aberration Database (Signature Genomics, Spokane, WA, USA), internal laboratory database at TYH and public databases [Database of Genomic Variant (DGV), International Standards for Cytogenomic Arrays Consortium Database (ISCA), Children Hospital of Philadelphia database (CHOP), Database of Chromosomal Imbalance and Phenotype in Humans using Ensembl Resources (DECIPHER)]. Categorization of CNVs was based on available phenotypes and comparison of phenotypes with genes in the region of copy gain or loss. This was done through searching Online Mendelian Inheritance in Man (OMIM), PubMed, RefSeq, the University of California Santa Cruz (UCSC) genome browser. [23] Confirmatory FISH/qPCR/conventional karyotype was performed as indicated. Parental testing was offered to aid further interpretation and classification. CNVs were classified as pathogenic, likely pathogenic, unknown/uncertain significance, or benign according to the 2011 American College of Medical Genetics (ACMG) practice guideline. [24] Only pathogenic and likely pathogenic CNVs are regarded as clinically significant.

### Management actions based on clinically significant CMA result

We identified 327 patients that fulfilled our inclusion criteria on whom we have performed CMA from the period January 2011 to May 2013. Retrospective medical record review was performed in September 2013 when all the abnormal CMA results had been disclosed to the patients/families in the post-test genetic counseling session. We analyzed the detection rate of clinically significant CMA results (pathogenic or likely pathogenic) and the medical management recommendations directly based on these findings. Since the interpretation of CNVs can evolve with new evidence over a short period of time, we also evaluated CNVs classified as “benign” and “unknown significance” for comparison.

A recommendation for clinical action was defined as any management recommendations prompted by CMA results including recurrent surveillance (S), specialist referral/assessment (R), diagnostic investigation (D) such as laboratory tests, ECG, diagnostic imaging studies etc., medical/surgical procedure (P), drug administration (M) (such as indication/contraindication for drug treatment), lifestyle recommendation (L) and other interventions (O) such as alternative therapies etc. [18], [19] Information on the clinically actionable genomic regions and the level of supporting evidence (Level 1 to 4) proposed by Riggs et al. [19] was used to analyze our findings. We did not include genetic counseling (for advice on reproductive options and/or prenatal diagnosis), confirmatory karyotype/QF-PCR/FISH, or parental testing which was done to clarify CNVs inheritance as countable clinical action.

### Statistical Analysis

Unpaired t-test was used for comparing CNVs size between pathogenic or likely pathogenic group and unknown clinical significance group. Fisher's exact tests were used to examine any potential association between CMA outcome and patients' characteristics including age group, gender and indications for CMA. Statistical analysis was carried out using IBM SPSS Statistics software version 19. A two-tailed p-value of less than 0.05 was treated as statistically significant.

### Ethics Statement

Approval was obtained from the Institutional Review Board of the University of Hong Kong and Hospital Authority of Hong Kong West Cluster. The title of the approved study is “Comparative study in prenatal/postnatal diagnostic detection using microarray technology and conventional cytogenetic analysis”, under the reference number UW10-226. Written informed consent was obtained from all parents/legal guardians.

## Results

Three hundred and twenty seven patients had CMA testing in the 29 months period and all were included in our analysis. Thirty-three patients were found to have pathogenic CNVs; 4 with likely pathogenic CNVs; 40 with CNVs of unknown significance, while the rest had benign CNVs. The detection rate of clinically significant CNVs (pathogenic or likely pathogenic) was 11.3% (37/327). In the group with clinically significant findings, 22 patients had copy number loss (deletions), 9 had copy number gain (duplications), and 6 patients had both deletion and duplication. There were a total of 45 clinical significant CNVs and 6 CNVs of unknown clinical significance found in these 37 patients. Of the group with CNVs of unknown clinical significance, 11 were deletions, 26 were duplications, and 3 were both deletion and duplication. There were a total of 45 CNVs of unknown clinical significance in these 40 patients. (See Table 1 for characteristics of the patients, Table 2 for CNVs types and numbers in clinically significant CNVs and CNVs with unknown significance group.)

**Table 1:** Patients' characteristics and CMA findings.

	[A] Number of patients with Pathogenic or Likely Pathogenic CNVs (%)	[B] Number of patients with CNVs of Unknown Clinical Significance (%)	[C] Number of patients with Benign CNVs (%)	[A] vs [B+C] comparison (by Fisher's exact test)
**Total**	37/327 (11.3%)	40/327 (12.2%)	250/327 (76.5%)	
**Age**				**p<0.001**
<12 m	20/37 (54.1%)	4/40 (10.0%)	34/250 (13.6%)	
1 – 5 y	10/37 (27.0%)	27/40 (67.5%)	164/250 (65.6%)	
6 – 10 y	2/37 (5.4%)	6/40 (15.0%)	28/250 (11.2%)	
11 – 18 y	4/37 (10.8%)	2/40 (5.0%)	20/250 (8.0%)	
>18 y	1/37 (2.7%)	1/40 (2.5%)	4/250 (1.6%)	
**Gender**				**p<0.001**
Male	15/37 (40.5%)	32/40 (80.0%)	172/250 (68.8%)	
Female	22/37 (59.5%)	8/40 (20.0%)	78/250 (31.2%)	
**Indications (Number of total cases)**				**p<0.001**
Neurodevelopmental disorders (DD/ID/ASD) (215 cases)	9/37 (24.3%)	28/40 (70.0%)	178/250 (71.2%)	
	9/215 (4.2%)*	28/215 (13.0%)*	178/215 (82.8%)*	
MCA/Dysmorphism ± neurodevelopmental disorders (105 cases)	26/37 (70.3%)	12/40 (30.0%)	67/250 (26.8%)	
	26/105 (24.8%)*	12/105 (11.4%)*	67/105 (63.8%)*	
Others (7 cases)	2/37 (5.4%)	0/40 (0%)	5/250 (2.0%)	
	2/7 (28.6%)*	0/7 (0%)*	5/7 (71.4%)*	

* =  detection rate based on referring indications. Abbreviations: MCA = Multiple Congenital Anomalies; DD = Developmental Delay; ID = Intellectual Disability; ASD = Autism Spectrum Disorders; m = months old, y = years old.

**Table 2:** CNVs type in patients with clinically significant CNVs and patients with CNVs of unknown clinical significance.

CNVs type	Patients with clinically significant CNVs (n = 37)	Patients with CNVs of unknown clinical significance (n = 40)
One deletion	21/37	11/40
Two deletions	1/37	0
One duplication	8/37	24/40
Two duplications	1/37	2/40
Deletion and Duplication	6/37	3/40

Patients with clinically significant CNVs were younger (age <12 months old, p<0.001, by Fisher's exact test), more likely to be female (p<0.001, by Fisher's exact test) and also more frequently had MCA/dysmorphism as indications for CMA (p<0.001, by Fisher's exact test), compared to others (Table 1). The mean size of clinical significant CNVs (9.20 Mb±4.56 Mb, mean±95% C.I.) was larger than that of CNVs of unknown significance (0.53 Mb±0.19 Mb) (p<0.001, by unpaired t-test). Copy number loss was found more frequently in clinically significant CNVs than in CNVs of unknown clinical significance (64.4% compared to 31.1%, p = 0.003, by Fisher's exact test).

Within the group of patients with clinically significant CNVs, there were patients with well-known genomic disorders including 1p36 deletion (n = 1), Wolf-Hirschhorn syndrome (n = 2), Cri du Chat syndrome (n = 2), Klinefelter syndrome (n = 1), 22q11.2 deletion (n = 2), and Williams syndrome (n = 3). CMA was offered to these patients either because their clinical features did not allow definitive diagnosis of the condition, or because they were atypical deletions or duplications that could not be detected by standard cytogenetic methods e.g. 22q11.2 deletion. [25] A few patients with interesting clinical/CMA findings in this cohort have been reported previously. [26]–[28]

Recommendations for clinical management were made in 75.7% (28 out of 37) patients with significant CNVs (Table 3), and in 2.5% (1 in 40) patients with unknown significance (see discussion section for detail of this case) respectively (p<0.001 by Fisher's exact test). Specific clinical actions for the patients with significant CNVs include 19 recommendations for surveillance (S), 24 specialist referrals (R), 25 diagnostic tests (D), 7 medical/surgical procedures (P), 15 recommendations regarding drug administration (M), 12 recommendations for lifestyle modification (L). According to the criteria by Riggs et al. [19], in nine of these patients, recommendations were based on Level 1 evidence, i.e. from practice guideline endorsed by a professional society; 10 were based on Level 2 evidence, i.e. from peer-reviewed publication describing medical management recommendations; 8 were based on Level 3 evidence, i.e. from peer-reviewed publications not regarding management but implying potential management based on clinical judgment; 1 was based on Level 4 evidence, i.e. could be managed symptomatically regardless of underlying diagnosis. Of the 28 patients with recommendations made based on the CMA result, 21 of them have findings overlapping with the clinically actionable genomic regions reported by Riggs et al. in 2013. [19] In the other 7 patients, management recommendations were made for one patient with Klinefelter syndrome (Level 1 evidence), one with trisomy X syndrome (Level 2 evidence) while in the rest the recommendations were based on case series/case reports (Level 3 evidence). The overall diagnostic yield of clinically actionable abnormal CMA findings is 8.6% (28/327).

**Table 3:** Management recommendations for clinically significant CNVs and recommendation according to the level of evidence.

Case no.	Age/Sex	Indication	Genomic coordinates (hg18) of CNVs	CNV size and type/syndrome or locus	Parental Testing	Clinical action	Level of evidence
1*	1m/F	MCA/dys	chr1: 825,513–6,489,818	5.6 Mb terminal copy loss at 1p36.33–p36.31/1p36 deletion	De novo	R,D,S,M	level 2
2*	24y/F	MCA/dys +DD	chr1: 825,513–3,930,371; chr10: 129,188,065–135,253,240	3.1 Mb copy gain at 1p36.33–p36.32 and 6 Mb copy loss at 10q26.2–q26.3/unbalanced translocation	N	no	no
3*	2m/F	MCA	chr1: 241,821,041–247,174,728	5.3 Mb terminal copy loss at 1q44/1q44 deletion	N	R,D	level 3
4*	18y/M	MCA+DD, cytogenetic diagnosis	chr3: 76,277–8,720,170; chr10: 102,474,001–135,246,402	8.6 Mb terminal copy loss at 3p26.3–p25.3; 32.7 Mb terminal copy gain at 10q24.31–q26.3/unbalanced translocation	N	no	no
5*	3y/F	MCA+cytogenetic diagnosis	chr4: 33,860–15,640,617	15.6 Mb copy loss at 4p16.3–p15.32/Wolf–Hirschhorn syndrome	N	R,D,P,M	level 2
6*	4m/M	MCA	chr5: 108,368–133,485; chr15: 20,372,901–37,603,955	25.1 kb copy loss (UCS) at 5p15.33; 17.2 Mb copy loss at 15q11.2–q14, karyotyping showed loss of chromosome 15 segment proximal to 15q15: karyotype 45,XY,der(5)t(5;15)(p15.3;q15)dn, –15/Expanded Prader–Willi syndrome	De novo	R,D,S,M,L for PWS; R,D for 15q13.3 del	level 1 for PWS, level 2 for 15q13.3 del
7*	6d/F	MCA	chr5: 108,467–17,723,107	17.6 Mb terminal copy loss at 5p15.33–p15.1/Cri du Chat syndrome	De novo	R,D,S	level 2
8*	7m/F	MCA	chr5: 108,467–1,237,565; chr5: 1,255,929–27,782,119	1.13 Mb terminal copy gain at 5p15.33; 26.5 Mb copy loss at 5p15.33–p14.1/Cri du Chat syndrome	N	R,D,S	level 2
9*	10m/F	MCA+DD+ASD	chr5: 108,467–1,597,323; chr11: 115,190,302–134,434,130	1.5 Mb terminal copy loss at 5p15.33; 19.2 Mb terminal copy gain at 11q23.2–q25/unbalanced mat. translocation t(5;11)(p15.3;q23)	Mat	R,D,S	level 2
10*	2m/F	MCA	chr5: 58,860,944–59,124,691; chr7: 72,382,850–73,776,237	263.7 kb copy loss (UCS) at 5q11.2–q12.1; 1.4 Mb copy loss at 7q11.23/Williams syndrome	N	R,D,S,M,L	level 1
11∧	3.1y/M	ASD	chr6: 162,541,977–163,015,824	473 kb copy gain at 6q26	De novo	no	no
12*	9m/F	MCA	chr7: 72,382,850-73,776,237	1.4 Mb copy loss at 7q11.23/Williams syndrome	N	R,D,S,M,L	level 1
13*	6d/M	MCA	chr7: 72,382,850–73,776,237; chr20: 34,118,917–34,173,592	1.4 Mb copy loss at 7q11.23; 54.6 kb copy loss (UCS) at 20q11.23/Williams syndrome	N	R,D,S,M,L	level 1
14*	5y/M	ASD	chr7: 110,765,432–111,124,405; chr15: 82,433,250–89,427,223	358.9 kb copy loss (paternal) at 7q31.1; 6.9 Mb copy loss at 15q25.2–q26.1/15q deletion	De novo	no	no
15*	6y/F	MCA+DD	chr9: 199,254–1,532,084; chr9: 1,544,692–29,980,935	1.3 Mb terminal copy loss at 9p24.3; 28.4 Mb copy gain at 9p24.3–p21.1/complex imbalanced 9p: karyotype 46,XX,der(9)(p21.1–>p24.3::p24.3–>qter)dn	De novo	no	no
16*	2m/F	MCA	chr9: 95,929,405–96,708,956; chr22: 46,600,315–49,522,658	779.5 kb copy gain (UCS) at 9p22.32; 2.9 Mb copy loss at 22q13.31–q13.33/ring chr22 with 22q13 microdeletion (including SHANK3)	N	R,D,S,L	level 3
17*	4y/F	MCA+DD	chr10: 125,911,563–135,253,240	9.3 Mb terminal copy loss at 10q26/10q26 deletion	De novo	D	level 3
18*	2m/M	MCA	chr16: 14,957,300–16,195,404	1.2 Mb copy loss at 16p13.11/16p13.11 microdeletion	N	R,D,P	level 3
19∧	2.5y/M	ASD	chr16: 15,033,259–16,195,404	1.2 Mb copy gain at 16p13.11/16p13.11 duplication	Mat	D	level 3
20∧	8y/M	ASD+DD	chr16: 15,033,259–16,195,404	1.2 Mb copy gain at 16p13.11/16p13.11 duplication	N	D	level 3
21*	1y/M	ASD	chr16: 28,395,992–28,953,785	558 kb copy loss at 16p11.2/16p11.2 (SH2B1 gene) microdeletion	De novo	R,S	level 2
22*	11y/M	Dys+DD	chr16: 54,476,646–58,816,939	4.3 Mb copy loss at 16q12.2–q21/16q12.2 deletion	De novo	D	level 4
23*	1m/M	MCA/dys	chr17: 740,287–1,530,746	790 kb copy gain at 17p13.3/17p13.3 duplication	N	R,S	level 3
24*	7m/M	MCA	chr17: 2,520,702–3,680,586	1.16 Mb copy loss at 17p13.3–p13.2/17p13.3p13.2 (LIS1 intragenic deletion)	De novo	no	no
25*	4m/F	MCA/dys	chr17: 26,140,621–27,346,744	1.2 Mb copy gain at 17q11.2/17q11.2 NF1 duplication	N	R,S	level 3
26*	8m/F	MCA	chr17: 43,878,156–45,719,328; chr17: 62,047,278–62,372,365	1.8 Mb copy loss at 17q21.32–q21.33; 325 kb copy gain (UCS) at 17q24.2/17q21.32–q21.33 deletion	N	R,D,P,M,L	level 2
27*	2m/F	MCA+cytogenetic diagnosis	chr18: 30,273,585–62,939,673	32.6 Mb copy gain at 18q12.1–q22.1/inverted duplication 18q12.1–q22.1	Only mother tested, not mat.	no	no
28*	1m/F	MCA	chr22: 17,299,469–19,790,658	2.5 Mb copy loss at 22q11.21/22q11 deletion	De novo	R,D,S,M,L	level 1
29*	3y/M	ASD	chr22: 17,299,469–19,790,658	2.5 Mb copy loss at 22q11.21/22q11 deletion	De novo	R,D,S,M,L	level 1
30∧	4.9y/M	ASD	chrX: 22,857,404–22,980,069	122 kb copy loss at Xp22.11 involving deletion of the DDX53 gene	Mat	no	no
31*	2m/F	MCA, cytogenetic diagnosis	chrX: 71,010,717–154,881,514	83.9 Mb terminal copy gain at Xq13.1–q28/functional partial monosomy 15 and Trisomy Xq, karyotype 46,XX,der(15)t(X;15)(q13.1;p10)dn	N	R,D,S,M,L	level 2
32*	13y/F	other	chrX: 107,539,632–107,750,773; chrX: 107,759,164–108,180,837	211.1 kb copy loss at Xq22.3; 421.6 kb copy gain at Xq22.3/X–linked Alport plus diffuse leiomyomatosis syndrome	Only mother tested, not mat.	R,D,S,M	level 2
33*	9m/F	Dys+DD+cytogenetic diagnosis	chrX: 134,459,007–134,901,914; chr4: 33,860–11,295,959	442.9 kb copy loss (UCS) at Xq26.3; 11.2 Mb terminal copy loss at 4p16.3–p15.33/Wolf–Hirschhorn syndrome	De novo	R,D,P,M	level 2
34*	3y/F	ASD	chrX: 216,519–4,031,220	3.8 Mb copy loss at Xp22.33/Xp22.33 (SHOX) deletion Léri–Weill dyschondrosteosis syndrome	N	R,D,P,S,M,L	level 1
35*	12y/F	other, genetic diagnosis	chrX: 216,519–39,315,013	39.1 Mb terminal copy loss at Xp22.33–p11.4/Xp22.33p11.4	De novo	R,D,P,S,M,L	level 1
36*	9m/F	MCA+DD	chrX: 369,190–886,653; 18: 3,404,569–3,886,147	517.4 kb copy gain (maternal) at Xq28; 481.5 kb copy gain at 18p11.31/18p11.31 duplication	Mat	no	no
37*	3y/M	ASD	chrX	47,XXY (gain of chrX)/Klinefelter syndrome	Inconclusive; Karyotype of mother: mos 47,XXX[1]/46,XX[29]	R,D,P,S,M,L	level 1

Abbreviations: MCA = Multiple Congenital Anomalies; DD = Developmental Delay; ASD = Autism Spectrum Disorders; Dys = Dysmorphism; UCS = Uncertain Clinical Significance.

Abbreviations: S = Surveillance; R =  specialist Referral/assessment; D = Diagnostic testing; P = medical/surgical Procedure; M = Medication administration; L = Lifestyle recommendation; O = Other interventions.

* = pathogenic CNVs, ∧ = likely pathogenic CNVs.

N = not tested; Mat  =  maternal inheritance; Pat  =  paternal inheritance.

## Case Illustration

### Level 1 evidence for clinical action: 47,XXY/Klinefelter syndrome (Case 37 in Table 3)

A 3 year old boy presented with speech delay and autistic features. He was born preterm following a spontaneous monochorionic diamniotic twin pregnancy. CMA showed arr(1-22,X)×2,(Y)×1 (confirmed by karyotype). He was referred to the endocrinology clinic, where he was managed according to existing protocols for Klinefelter syndrome with other various recommendations (R,D,P,S,M,L). [29], [30] His otherwise healthy twin was also confirmed to have Klinefelter Syndrome. Their mother was pregnant when the diagnosis of Klinefelter syndrome was disclosed and parental karyotype was offered due to their anxiety. The karyotype of their father was normal while that of their mother (30 years old) showed low level mosaicism of 47,XXX[1]/46,XX[29]. Sex chromosome aneuploidy is recognized to be a normal phenomenon in culture lymphocytes from women of different ages and specifically it was reported that 4% of women between 23 to 34 years of age can have X chromosome gain. [31] This low frequency of aneuploid cells does not signify an increased risk of prenatal diagnosis of sex chromosomal aneuploidy in the fetus and this was explained to the parents in subsequent session of genetic counseling.

### Level 2 evidence for clinical action: 1p36 deletion (Case 1 in Table 3)

A newborn girl was diagnosed to have Ebstein anomaly. She developed a generalized seizure shortly after cardiac surgery on day 3 of life. CMA showed diagnosis of 1p36 microdeletion syndrome (OMIM #607872). Neurodevelopmental and feeding assessment (R), eye assessment (S), EEG (D), brain MRI (D), USG kidney (D), and thyroid function test (S) were recommended. [32] Thyroxine replacement (M) and antiepileptic therapy (M) were prescribed subsequently. Parents were provided with extensive counseling on the prognosis and management of the condition. The patient had a prolonged hospital stay and died at 20 months of age after an acute deterioration without identifiable cause.

### Level 3 evidence for clinical action: 1q44 deletion (Case 3 in Table 3)

A full term baby with pulmonary atresia, ventricular septal defect and thyroid agenesis was referred for genetic evaluation at 2 months of age. She had a history of intrauterine growth restriction and exhibited failure to thrive. CMA showed 1q44 deletion [arr 1q44(241,821,041–247,174,728) ×1]. Her clinical features were consistent with the phenotype associated with 1q44 deletion (OMIM #612337). Seizures and abnormal corpus callosum are commonly reported. [33]–[35] Our patient was recommended to have brain MRI which showed a hypoplastic corpus callosum. Upon our recommendation, she was followed by the neurologists and confirmed to have severe DD. She later developed seizures and required antiepileptic treatment.

### No recommendation for clinical action: submicroscopic unbalanced translocation (Case 2 in Table 3)

A 24 year-old female being followed in the paediatric clinic was referred for evaluation of developmental delay and dysmorphic features. She had a past history of being small for gestational age, short stature, scoliosis, hypotonia and resolved tremor/head shaking. All her previous investigations, including brain MRI (hypoplastic inferior vermis), karyotype, FISH for Williams syndrome, 7 blood tests and 2 urine tests for metabolic diseases, spine MRI, nerve conduction velocity/electromyography, Tensilon test, muscle biopsy, were non-diagnostic. CMA showed terminal 1p36.33p36.32 duplication and terminal 10q26.2q26.3 deletion, suggesting an unbalanced translocation. The unbalanced translocation was then confirmed by FISH. Patients with 10q26 deletion are reported to share similar features of ID/DD, dysmorphic features, as well as behavioral problems. [36]–[38] Although there was no clinical action prompted in this patient, this case showed how first-tier CMA testing might have avoided 15 unnecessary investigations (including the invasive muscle biopsy) and ended the diagnostic odyssey.

## Discussion

A growing body of evidence demonstrates the superior diagnostic yield of CMA compared to conventional karyotype, and CMA has been endorsed by various professional organizations as a first-tier investigation for children with unexplained DD/ID/ASD and/or MCAs. [2], [3] However in the States, the evidence has not been sufficient to support coverage of CMA by many health insurance providers. The decision of which often depends on the evidence of whether a test will influence medical management and result in improvement in health outcome. In Hong Kong where most medical expenses are publicly funded, similar decisions have to be made by the government for supporting new testing and therefore it is also our interest to study whether a genetic test can significantly affect clinical management and patient outcome.

To evaluate the degree to which CMA can impact clinical management of our patient population, we retrospectively reviewed the medical records of 327 patients tested in a single laboratory and managed in a single paediatric unit (2 teaching hospitals) over a 29-month period. We included only patients in whom the CMA results had been disclosed and management recommendations have been suggested in a post-test genetic counseling session. We had full access to the medical records of all patients and the latest recruited patient had at least 3-month follow-up period for the evaluation of outcome after the recommendation. It was noted that parental testing rate was 56.8% in the 37 patients with significant CMA results, due to either parental refusal.

Compared to 4 previous studies on clinical utility of CMAs [18]–[21], we found a detection rate of 8.6% for clinically actionable CNVs, which was comparable if not slightly higher than the reported number of 3.6%∼7% (Table 4). There are significant differences in the design in previous studies and we have adopted the approach used by Riggs et al. [19] We grouped the recommendations into various categories (S, R, D, P, M, L, O) and linked them to the current level of evidence. This standardized approach allows better comparison with other studies and re-evaluation of our own finding with new evidence after a certain period of time. [39]

**Table 4:** Comparison of clinically actionable abnormal CMA findings in published studies.

Study (published year)	Coulter et al (2011)	Ellison et al (2012)	Riggs et al (2014)	Henderson et al (2014)	Our study
Study design	Retrospective review of medical records	Retrospective laboratory database review (Signature Genomics)	Retrospective laboratory database review (ISCA)	Retrospective review of electronic medical records	Retrospective review of medical records
Number of subjects	1,792	46,298	28,256	1,780	327 (first-tiered testing and with specific indications)
Study period	1 y (2009–2010)	7.5 y (2004–2011)	As in March 2012	3 y (2009–2012)	29m (2010–2013)
CMA platforms used	Not specified	Multiple BAC-based and oligo-arrays. (only those with oligo-arrays counted to evaluate clinical actionability)	Multiple platforms (not specified)	2 high resolution SNP array platforms	NimbleGen 135k oligonucleotide array
Definition of clinically actionable results	Findings that prompt specialist referral, imaging, diagnostic test or medication prescription.	1. Established microdeletion/microduplication syndromes; 2. Conditions with increased cancer susceptibility; 3. Other actionable conditions associated with dosage-sensitive genes.	Conditions diagnosable by CMA for which referral, diagnostic testing, surgical/interventional procedure, surveillance, medical and lifestyle changes would be recommended. The recommendations were stratified according to the level of evidence.	Findings that prompt recommendations of further action such as pharmacologic treatment, cancer-related screening, contraindications, additional evaluation or referrals.	Criteria by Riggs et al.
Diagnostic yield of significant results	235/1,792 (13.0%)	15.4% for the oligo-array (based on previous study from the same group)	4,125/28,256 (14.6%)	227/1,780 (12.7%)	37/327 (11.0%)
Clinical actionability in those with significant CMA results (%)	34.0–54.0%	35.0%	46.0% (66.0% for deletion cases and 11.0% for duplication cases)	54.7% (42.1% for patients referred for isolated neurodevelopmental disorders)	75.7% (44.0% for patients referred for isolated neurodevelopmental disorders)
Clinically actionability in the whole cohort (%)	3.6%	5.4%	7.0%	5.4%	8.6%

We observed a good correlation between the clinical significance and the clinical actionability of the CMA findings. Clinical management changes were recommended in 75.7% of patients with clinically significant CNVs; in 2.5% of patients with CNVs of unknown significance and in 0% with benign findings respectively. The clinically significant findings are larger in size and were more likely to be deletions than duplications. Interestingly, in those with clinically significant CNVs, management recommendations were made in 22/26 patients (84.6%) with MCA/dysmorphism as an indication for CMA, compared to 4/9 patients (44%) in those with neurodevelopmental disorders only. This lower clinical actionability rate for CNVs found in patients with isolated neurodevelopmental disorders was also observed in previous studies. [18], [21] Young age was associated with clinical significant CNVs in our study, which was also observed in the study of Coulter et al (2011). [18]

Only 1 investigation was ordered in 1 patient with CNV of unknown significance, referred for non-syndromic ASD. The recommendation (an ECG) was based on the finding of the same 20p12.3 duplication in a single case report on a patient with familial Wolf-Parkinson-White syndrome. [40] Parents agreed for ECG on the child because the non-invasive nature of the investigation and the availability of potential treatment if WPW was identified. In this patient, the ECG was normal. Similarly we have made a level 4 recommendation (brain MRI) for another patient with DD, epilepsy and a 16p12 deletion based on positive finding of bilateral frontoparietal polymicrogyria (BFPP) in a patient reported with a similar deletion. [41] In our patient, brain MRI did not show BFPP. This illustrates that with our study design we are able to evaluate not only the recommendations we made based on the CMA findings but the actual clinical outcomes of patients. This will be important especially for CNVs with clinical actions based on low level of evidence.

There are multiple advantages of reaching a genetic diagnosis in patients. First of all, making a diagnosis allows estimation of recurrence risk and informed decisions about future pregnancies for the parents. As illustrated by case 37 (with Level 1 evidence), the clinical utility of CMA in the proband can extend beyond the affected individuals and familial testing can reveal diagnosis in a sibling (normal development but with Klinefelter syndrome) with less obvious clinical manifestations. Secondly, for some like patient 2, a diagnosis of submicroscopic unbalanced translocation helps to end the 24 years of diagnostic odyssey. If the test is being offered as first-tier testing in a similar patient at the current time, it may help to avoid a lot of unnecessary investigations including the more invasive ones. The direct benefits to clinical management have been demonstrated by previous studies. [18]–[21] Our study was able to confirm that a significant CMA finding influenced medical management in 75% of our patients. Although our study involves a smaller number of patients, we were able to study at least the short-term direct clinical outcomes in our patients who have received recommendations based on their CMA findings. Future goals will be the long-term study of the impact on clinical outcomes for a larger cohort of patients with significant CMA findings and how the changing interpretation of CMA over time may change the clinical management of patients with different categories of findings. We advocate the use of diagnostic yield of clinically actionable results in the evaluation of CMA testing as this allows the clinicians to consider both clinical validity and clinical utility of CMA under the ACCE framework [42]–[47] and it provides a link between the practice of medical genetics and evidence-based medicine.

## References

[1] Hochstenbach R, van Binsbergen E, Engelen J, Nieuwint A, Polstra A, et al (2009) Array analysis and karyotyping: workflow consequences based on a retrospective study of 36,325 patients with idiopathic developmental delay in the Netherlands. Eur J Med Genet 52: 161–169. 19362174 10.1016/j.ejmg.2009.03.015

[2] Miller DT, Adam MP, Aradhya S, Biesecker LG, Brothman AR, et al (2010) Consensus statement: chromosomal microarray is a first-tier clinical diagnostic test for individuals with developmental disabilities or congenital anomalies. Am J Hum Genet 86: 749–764. 20466091 10.1016/j.ajhg.2010.04.006 PMC2869000

[3] Manning M, Hudgins L (2010) Array-based technology and recommendations for utilization in medical genetics practice for detection of chromosomal abnormalities. Genet Med 12: 742–745. 20962661 10.1097/GIM.0b013e3181f8baad PMC3111046

[4] Sagoo GS, Butterworth AS, Sanderson S, Shaw-Smith C, Higgins JP, et al (2009) Array CGH in patients with learning disability (mental retardation) and congenital anomalies: updated systematic review and meta-analysis of 19 studies and 13,926 subjects. Genet Med 11: 139–146. 19367186 10.1097/GIM.0b013e318194ee8f

[5] Shen Y, Dies KA, Holm IA, Bridgemohan C, Sobeih MM, et al (2010) Clinical genetic testing for patients with autism spectrum disorders. Pediatrics 125: e727–735. 20231187 10.1542/peds.2009-1684 PMC4247857

[6] Edelmann L, Hirschhorn K (2009) Clinical utility of array CGH for the detection of chromosomal imbalances associated with mental retardation and multiple congenital anomalies. Ann N Y Acad Sci 1151: 157–166. 19154522 10.1111/j.1749-6632.2008.03610.x

[7] Stankiewicz P, Beaudet AL (2007) Use of array CGH in the evaluation of dysmorphology, malformations, developmental delay, and idiopathic mental retardation. Curr Opin Genet Dev 17: 182–192. 17467974 10.1016/j.gde.2007.04.009

[8] Kang JU, Koo SH (2012) Evolving applications of microarray technology in postnatal diagnosis (review). Int J Mol Med 30: 223–228. 22580383 10.3892/ijmm.2012.988

[9] Battaglia A, Doccini V, Bernardini L, Novelli A, Loddo S, et al (2013) Confirmation of chromosomal microarray as a first-tier clinical diagnostic test for individuals with developmental delay, intellectual disability, autism spectrum disorders and dysmorphic features. Eur J Paediatr Neurol 17: 589–599. 23711909 10.1016/j.ejpn.2013.04.010

[10] Wu Y, Ji T, Wang J, Xiao J, Wang H, et al (2010) Submicroscopic subtelomeric aberrations in Chinese patients with unexplained developmental delay/mental retardation. BMC Med Genet 11: 72. 20459802 10.1186/1471-2350-11-72 PMC2892449

[11] Lee CG, Park SJ, Yun JN, Ko JM, Kim HJ, et al (2013) Array-based comparative genomic hybridization in 190 Korean patients with developmental delay and/or intellectual disability: a single tertiary care university center study. Yonsei Med J 54: 1463–1470. 24142652 10.3349/ymj.2013.54.6.1463 PMC3809862

[12] Park SJ, Jung EH, Ryu RS, Kang HW, Ko JM, et al (2011) Clinical implementation of whole-genome array CGH as a first-tier test in 5080 pre and postnatal cases. Mol Cytogenet 4: 12. 21549014 10.1186/1755-8166-4-12 PMC3114015

[13] Byeon JH, Shin E, Kim GH, Lee K, Hong YS, et al (2014) Application of array-based comparative genomic hybridization to pediatric neurologic diseases. Yonsei Med J 55: 30–36. 24339284 10.3349/ymj.2014.55.1.30 PMC3874920

[14] Saam J, Gudgeon J, Aston E, Brothman AR (2008) How physicians use array comparative genomic hybridization results to guide patient management in children with developmental delay. Genet Med 10: 181–186. 18344707 10.1097/GIM.0b013e3181634eca

[15] Mroch AR, Flanagan JD, Stein QP (2012) Solving the puzzle: case examples of array comparative genomic hybridization as a tool to end the diagnostic odyssey. Curr Probl Pediatr Adolesc Health Care 42: 74–78. 22325475 10.1016/j.cppeds.2011.10.003

[16] Adams SA, Coppinger J, Saitta SC, Stroud T, Kandamurugu M, et al (2009) Impact of genotype-first diagnosis: the detection of microdeletion and microduplication syndromes with cancer predisposition by aCGH. Genet Med 11: 314–322. 19365269 10.1097/GIM.0b013e3181a028a5

[17] Adam MP, Justice AN, Schelley S, Kwan A, Hudgins L, et al (2009) Clinical utility of array comparative genomic hybridization: uncovering tumor susceptibility in individuals with developmental delay. J Pediatr 154: 143–146. 19187739 10.1016/j.jpeds.2008.07.045 PMC4072200

[18] Coulter ME, Miller DT, Harris DJ, Hawley P, Picker J, et al (2011) Chromosomal microarray testing influences medical management. Genet Med 13: 770–776. 21716121 10.1097/GIM.0b013e31821dd54a

[19] Riggs ER, Wain KE, Riethmaier D, Smith-Packard B, Faucett WA, et al (2014) Chromosomal microarray impacts clinical management. Clin Genet 85: 147–153. 23347240 10.1111/cge.12107

[20] Ellison JW, Ravnan JB, Rosenfeld JA, Morton SA, Neill NJ, et al (2012) Clinical utility of chromosomal microarray analysis. Pediatrics 130: e1085–1095. 23071206 10.1542/peds.2012-0568

[21] Henderson LB, Applegate CD, Wohler E, Sheridan MB, Hoover-Fong J, et al.. (2014) The impact of chromosomal microarray on clinical management: a retrospective analysis. Genet Med. 10.1038/gim.2014.18 24625444

[22] Regier DA, Friedman JM, Marra CA (2010) Value for money? Array genomic hybridization for diagnostic testing for genetic causes of intellectual disability. Am J Hum Genet 86: 765–772. 20398885 10.1016/j.ajhg.2010.03.009 PMC2869008

[23] Kan AS, Lau ET, Tang WF, Chan SS, Ding SC, et al (2014) Whole-Genome Array CGH Evaluation for Replacing Prenatal Karyotyping in Hong Kong. PLoS One 9: e87988. 24505343 10.1371/journal.pone.0087988 PMC3914896

[24] Kearney HM, Thorland EC, Brown KK, Quintero-Rivera F, South ST (2011) American College of Medical Genetics standards and guidelines for interpretation and reporting of postnatal constitutional copy number variants. Genet Med 13: 680–685. 21681106 10.1097/GIM.0b013e3182217a3a

[25] Verhagen JM, Diderich KE, Oudesluijs G, Mancini GM, Eggink AJ, et al (2012) Phenotypic variability of atypical 22q11.2 deletions not including TBX1. Am J Med Genet A 158a: 2412–2420. 22893440 10.1002/ajmg.a.35517

[26] Ho AC, Liu AP, Lun KS, Tang WF, Chan KY, et al (2012) A newborn with a 790 kb chromosome 17p13.3 microduplication presenting with aortic stenosis, microcephaly and dysmorphic facial features - is cardiac assessment necessary for all patients with 17p13.3 microduplication? Eur J Med Genet 55: 758–762. 23063769 10.1016/j.ejmg.2012.09.011

[27] Liu AP, Tang WF, Lau ET, Chan KY, Kan AS, et al (2013) Expanded Prader-Willi syndrome due to chromosome 15q11.2–14 deletion: report and a review of literature. Am J Med Genet A 161a: 1309–1318. 23633107 10.1002/ajmg.a.35909

[28] Tsang JS, Tong DKH, Chung BHY, Tang MHY, Lau ET, et al (2013) Alport's syndrome: case of a giant esophageal tumor. Esophagus 10: 114–117.

[29] Aksglaede L, Link K, Giwercman A, Jorgensen N, Skakkebaek NE, et al (2013) 47,XXY Klinefelter syndrome: clinical characteristics and age-specific recommendations for medical management. Am J Med Genet C Semin Med Genet 163c: 55–63. 23345262 10.1002/ajmg.c.31349

[30] Groth KA, Skakkebaek A, Host C, Gravholt CH, Bojesen A (2013) Clinical review: Klinefelter syndrome-a clinical update. J Clin Endocrinol Metab 98: 20–30. 23118429 10.1210/jc.2012-2382

[31] Nowinski GP, Van Dyke DL, Tilley BC, Jacobsen G, Babu VR, et al (1990) The frequency of aneuploidy in cultured lymphocytes is correlated with age and gender but not with reproductive history. Am J Hum Genet 46: 1101–1111. 2339703 PMC1683821

[32] (1993) GeneReviews. Seattle (WA): University of Washington, Seattle.

[33] Orellana C, Rosello M, Monfort S, Oltra S, Quiroga R, et al (2009) Corpus callosum abnormalities and the controversy about the candidate genes located in 1q44. Cytogenet Genome Res 127: 5–8. 20110648 10.1159/000279261

[34] van Bon BW, Koolen DA, Borgatti R, Magee A, Garcia-Minaur S, et al (2008) Clinical and molecular characteristics of 1qter microdeletion syndrome: delineating a critical region for corpus callosum agenesis/hypogenesis. J Med Genet 45: 346–354. 18178631 10.1136/jmg.2007.055830

[35] Caliebe A, Kroes HY, van der Smagt JJ, Martin-Subero JI, Tonnies H, et al (2010) Four patients with speech delay, seizures and variable corpus callosum thickness sharing a 0.440 Mb deletion in region 1q44 containing the HNRPU gene. Eur J Med Genet 53: 179–185. 20382278 10.1016/j.ejmg.2010.04.001

[36] Iourov IY, Vorsanova SG, Kurinnaia OS, Zelenova MA, Silvanovich AP, et al (2012) Molecular karyotyping by array CGH in a Russian cohort of children with intellectual disability, autism, epilepsy and congenital anomalies. Mol Cytogenet 5: 46. 23272938 10.1186/1755-8166-5-46 PMC3547809

[37] Courtens W, Wuyts W, Rooms L, Pera SB, Wauters J (2006) A subterminal deletion of the long arm of chromosome 10: a clinical report and review. Am J Med Genet A 140: 402–409. 16419133 10.1002/ajmg.a.31053

[38] Lukusa T, Fryns JP (2000) Pure distal monosomy 10q26 in a patient displaying clinical features of Prader-Willi syndrome during infancy and distinct behavioural phenotype in adolescence. Genet Couns 11: 119–126. 10893663

[39] Palmer E, Speirs H, Taylor PJ, Mullan G, Turner G, et al (2014) Changing interpretation of chromosomal microarray over time in a community cohort with intellectual disability. Am J Med Genet A 164a: 377–385. 24311194 10.1002/ajmg.a.36279

[40] Mills KI, Anderson J, Levy PT, Cole FS, Silva JN, et al (2013) Duplication of 20p12.3 associated with familial Wolff-Parkinson-White syndrome. Am J Med Genet A 161a: 137–144. 23239491 10.1002/ajmg.a.35701

[41] Borgatti R, Marelli S, Bernardini L, Novelli A, Cavallini A, et al (2009) Bilateral frontoparietal polymicrogyria (BFPP) syndrome secondary to a 16q12.1–q21 chromosome deletion involving GPR56 gene. Clin Genet 76: 573–576. 19807741 10.1111/j.1399-0004.2009.01262.x

[42] Hastings R, de Wert G, Fowler B, Krawczak M, Vermeulen E, et al (2012) The changing landscape of genetic testing and its impact on clinical and laboratory services and research in Europe. Eur J Hum Genet 20: 911–916. 22453292 10.1038/ejhg.2012.56 PMC3421130

[43] Burke W, Atkins D, Gwinn M, Guttmacher A, Haddow J, et al (2002) Genetic test evaluation: information needs of clinicians, policy makers, and the public. Am J Epidemiol 156: 311–318. 12181100 10.1093/aje/kwf055

[44] Haddow JE, Palomaki GE (2004) ACCE: a model process for evaluating data on emerging genetic tests. Human genome epidemiology: A scientific foundation for using genetic information to improve health and prevent disease: 217–233.

[45] Wald N, Cuckle H (1989) Reporting the assessment of screening and diagnostic tests. Br J Obstet Gynaecol 96: 389–396. 2751951 10.1111/j.1471-0528.1989.tb02411.x

[46] U.S. Department of Health and Human Services, Secretary's Advisory Committee on Genetic Testing (2000) Request for public comment on a proposed classification methodology for determining level of review for genetic tests. Federal Register 65: 76643–76645.

[47] Dierking A, Schmidtke J (2014) The future of Clinical Utility Gene Cards in the context of next-generation sequencing diagnostic panels. Eur J Hum Genet. 10.1038/ejhg.2014.23 PMC4200435 24549053

